# Machinery

**Published:** 1883-11

**Authors:** 


### MACHINERY.

In the current number will be found an article upon dental machinery, which we commend to the reader.

There has been a slight hesitation in speaking freely upon such subjects in some of the journals, for fear the publishers, being interested in the sale of certain wares, might regard their business affairs as being interfered with.

The INDEPENDENT PRACTITIONER invites, for the benefit of its readers, honest criticism of all apparatus that is offered in the market, irrespective of its commercial relations or value.

